# Fruits and vegetables intake and bladder cancer risk: a pooled analysis from 11 case–control studies in the BLadder cancer Epidemiology and Nutritional Determinants (BLEND) consortium

**DOI:** 10.1007/s00394-024-03436-5

**Published:** 2024-06-05

**Authors:** Iris W. A. Boot, Anke Wesselius, Sylvia H. J. Jochems, Evan Y. W. Yu, Cristina Bosetti, Martina Taborelli, Stefano Porru, Angela Carta, Klaus Golka, Xuejuan Jiang, Mariana C. Stern, Eliane Kellen, Hermann Pohlabeln, Li Tang, Margaret R. Karagas, Zuo-Feng Zhang, Jack A. Taylor, Carlo La Vecchia, Maurice P. Zeegers

**Affiliations:** 1 Department of Epidemiology, Maastricht University, P. Debeyeplein 1, 6229 HA Maastricht, The Netherlands; 2 CAPHRI, Department of Epidemiology, Maastricht University Medical Centre+, Maastricht, The Netherlands; 3 School of Nutrition and Translational Research in Metabolism, Maastricht University, 6229 ER Maastricht, The Netherlands; 4 Key Laboratory of Environmental Medicine and Engineering of Ministry of Education, and Department of Epidemiology and Biostatistics, School of Public Health, Southeast University, Nanjing, 210009 China; 5 Unit of Cancer Epidemiology, Department of Oncology, IRCCS-Istituto di Ricerche Farmacologiche Mario Negri, Via Giuseppe La Masa 19, 20156 Milan, Italy; 6 Unit of Cancer Epidemiology, CRO Aviano National Cancer Institute, Aviano, PN Italy; 7 Department of Diagnostics and Public Health, Section of Occupational Health, University of Verona, Verona, Italy; 8 Interuniversity Research Center, Integrated Models for Prevention and Protection in Environmental and Occupational Health, MISTRAL, University of Brescia, University of Milano-Bicocca, University of Verona, Verona, Italy; 9 Leibniz Research Centre for Working Environment and Human Factors, Sektion Lebenswissenschaften, Dortmund, Germany; 10 Department of Preventive Medicine, University of Southern California, Los Angeles, CA USA; 11 Department of Ophthalmology, University of Southern California, Los Angeles, CA USA; 12 Leuven University Centre for Cancer Prevention (LUCK), Leuven, Belgium; 13 Leibniz Institute for Prevention Research and Epidemiology-BIPS, Bremen, Germany; 14 Department of Cancer Prevention and Control, Roswell Park Cancer Institute, Buffalo, NY USA; 15 Department of Epidemiology, Geisel School of Medicine at Dartmouth, Hanover, NH USA; 16 Departments of Epidemiology, UCLA Center for Environmental Genomics, Fielding School of Public Health, University of California, Los Angeles (UCLA), Los Angeles, CA USA; 17 Epidemiology Branch, and Epigenetic and Stem Cell Biology Laboratory, National Institute of Environmental Health Sciences, NIH, Research Triangle Park, NC USA; 18 Department of Clinical Medicine and Community Health, Università degli Studi di Milano, 20133 Milan, Italy

**Keywords:** Bladder cancer, Nutritional oncology, Pooled case control study, Fruits, Vegetables

## Abstract

**Purpose:**

High consumption of fruits and vegetables decrease the risk of bladder cancer (BC). The evidence of specific fruits and vegetables and the BC risk is still limited.

**Methods:**

Fruit and vegetable consumptions in relation to BC risk was examined by pooling individual participant data from case–control studies. Unconditional logistic regression was used to estimate study-specific odds ratio’s (ORs) with 95% confidence intervals (CIs) and combined using a random-effects model for intakes of total fruits, total vegetables, and subgroups of fruits and vegetables.

**Results:**

A total of 11 case–control studies were included, comprising 5637 BC cases and 10,504 controls. Overall, participants with the highest intakes versus the lowest intakes of fruits in total (OR 0.79; 95% CI 0.68–0.91), citrus fruits (OR 0.81; 95% CI 0.65–0.98), pome fruits (OR 0.76; 95% CI 0.65–0.87), and tropical fruits (OR 0.84; 95% CI 0.73–0.94) reduced the BC risk. Greater consumption of vegetables in total, and specifically shoot vegetables, was associated with decreased BC risk (OR 0.82; 95% CI 0.68–0.96 and OR 0.87; 95% CI 0.78–0.96, respectively). Substantial heterogeneity was observed for the associations between citrus fruits and total vegetables and BC risk.

**Conclusion:**

This comprehensive study provides compelling evidence that the consumption of fruits overall, citrus fruits, pome fruits and tropical fruits reduce the BC risk. Besides, evidence was found for an inverse association between total vegetables and shoot vegetables intake.

## Introduction

Consumption of fruits and vegetables is an important part of a healthy diet and has been linked to a reduced risk of cancer [1]. A report on diet and bladder cancer (BC) from the *WCRF Continuous Update Project* concluded that high intakes of fruit and vegetables could decrease the BC risk [2]. In addition, results of a large systematic review suggested that the consumption of citrus fruits and cruciferous vegetables decreases BC risk [3], and another review suggested that a diet rich in vegetables and fruits might be protective against BC [4].

Previous studies have often lacked adequate statistical power to detect associations between dietary factors and BC risk. Aside from total fruit and total vegetable consumption, associations between subgroups of fruits and vegetables and BC risk have been reported sporadically and may be subject to publication bias. As fruits and vegetables are heterogeneous with respect to phytochemical content [5], associations with BC risk may differ between types of fruits and vegetables. The use of individual data from multiple case–control studies could substantially increase statistical power, and covariates can be standardized across studies.

The aim of this large-scale pooled study was to investigate the association between total fruit and total vegetable consumption and specific subgroups of fruits and vegetables and BC risk using data of 5,648 BC cases and 10,517 controls from 11 studies which were included in the Bladder cancer Epidemiology and Nutritional Determinants (BLEND) consortium.

## Methods

### Study population

Data were analyzed from the BLEND study. BLEND is a large international nutritional consortium on BC, which includes nineteen case–control studies originating from countries all over the world [6]. Eleven of the nineteen case–control studies had sufficient information (i.e., data on usual dietary intake, method of dietary assessment, geographical region, ethnicity, gender, smoking status, disease status, age at BC diagnosis and/or age at enrollment of the study) to be eligible for inclusion in our study on the influence of total fruit and total vegetable consumption and specific subgroups of fruits and vegetables on BC risk. Studies originated from the USA, Belgium, Sweden, Italy, Canada and China. Each participating study has been approved by a local ethics committee. Informed consent was obtained from all individual participants included in each study.

### Data collection

Details on the methodology of the BLEND consortium have been described elsewhere [6]. Briefly, BC cases were ascertained by using medical record review or linkage with a cancer registry. Both non-muscle invasive BC (NMIBC) and muscle invasive BC (MIBC) were considered as outcomes.

For each study, participants were asked to report the frequency of fruit and vegetable items consumed during the preceding one or two years before study enrollment. All studies made use of a validated self-administered food frequency questionnaire (FFQ) or a questionnaire administered by a trained interviewer regarding the frequency of fruit and vegetable consumption. Summary details of the FFQs used in the included studies can be found in Appendix 1, Table 5. Total fruit consumption was computed as the sum of all fruit items in each study, and total vegetable consumption as the sum of all vegetable items provided. The following subgroups of fruits were defined: citrus fruits, pome fruits, soft fruits, stone fruits, tropical fruits, fruit mixtures, and fruit products (Table 1). For vegetables, the defined subgroups were: leaf vegetables, brassica, stalk vegetables, shoot vegetables, tubers, onion-family vegetables, root vegetables, fruit vegetables, pod seeds, fungi, seaweeds, vegetables mixtures, and vegetables products (Table 1). No analyses were performed for fruit mixtures, fruit products, onion-family vegetables, root vegetables, fruit vegetables, pod seeds, fungi, seaweeds, vegetable mixtures, or vegetable products because of the limited number of participants consuming fruits or vegetables from these subgroups.

**Table 1:** Characteristics of vegetable subgroups investigated in the pooled case–control analyses

Subgroups of fruits	
Citrus fruits	Lemons, oranges, tangerines, grapefruits, pomelos, limes, kumquats
Soft fruits	Strawberries, raspberries, white grapes, black grapes, loganberries, blackberries, dewberries, cloudberries, gooseberries, black currants, red currants, white currants, cranberries, bilberries, cowberries, blueberries, elderberries, rowanberries, physalis, mulberries, bearberries, sea buckthorns
Stone fruits	Apricots, peaches, nectarines, plums, damsons, mirabelles, greengages, sweet cherries, sour cherries, chickasaws, susinas, sloes, dates, lychees, persimmons, barbados cherries
Pome fruits	Apples, pears, quinces, medlars, and loquats
Tropical fruits	Bananas, pineapples, kiwi fruits, (water)melons, figs, mangos, pomegranates, passion fruits, cashew fruits, guavas, papayas, rose hips, sapodillas, carambolas, durians, jack fruits, chayotes, rambutans, tamarinds
Fruit products	Dried mixed fruits, mixed peels, glace cherries, crystallized pineapple, apple sauce, cranberry sauce
Fruit mixtures	Fruit cocktail, fruit salad

Subgroups of vegetables	
Leaf vegetables	Endive, lettuce, lamb’s lettuce, swiss chard, spinach, garden orache, cress seedling, mustard seedling, land cress, watercress, vine leaf, dandelion leaf, nettle, sorrel, purslane, parsley
Brassica	Broccoli, cauliflower, cabbage, red cabbage, Chinese cabbage, brussels sprouts, turnip tops, kohlrabi, curly kale
Stalk vegetables	Celery, fennel, sea kale, rhubarb
Shoot vegetables	Asparagus, chicory, globe artichoke, bamboo shoot, palm heart
Tubers	New potato, main crop potato, Jerusalem artichoke, sweet potato, yam, cassava, taro
Onion-family vegetables	Onion, spring onion, shallot, leek, garlic, chives
Root vegetables	Carrot, salsify, celeriac, parsnip, turnip, swede, radish, beetroot, parsley root
Fruit vegetables	Tomato (raw and cooked), aubergine, sweet pepper, chilli pepper, cucumber, courgette, cucurbita, other gourds, ackee, breadfruit, matoki, plantain, avocado, olive
Pod seeds	Pea, broad bean, wax bean, French bean, runner bean, sweet corn, okra
Fungi	Cultivated mushroom, field mushroom, honey mushroom, boletus, truffle, morel, cantharelle, orange agaric, oyster mushroom, shitake mushroom, straw mushroom
Seaweeds	Irish miss, kombu, laver, wakame
Vegetable mixes	Mixed vegetables, mustard and cress, pot herbs
Vegetable products	Mushy peas, garlic puree, tomato puree, vegetable puree, pickled gherkins, pickled onion, pickled red cabbage, sauerkraut

### Statistical analysis

Participants who reported a history of cancer other than nonmelanoma skin cancer prior to study entry, or had missing data on age at study entry, gender, smoking status, pack years, or fruit and vegetable consumption, were excluded from the analyses. In the analyses, fruit and vegetable consumption were categorized into three consumption levels (low intakes/moderate intakes/high intakes), corresponding to study-specific marginal tertiles. The investigated fruit subgroups (citrus fruits, pome fruits, soft fruits, stone fruits, and tropical fruits) and vegetable subgroups (leaf vegetables, brassica, stalk vegetables, shoot vegetables, and tubers) were also modeled as study-specific marginal tertiles. Although the tertile approach does not take into account true differences in the distribution of population intakes across studies, reported intakes may differ across studies based on country-specific portions sizes and differences in FFQs used, and is therefore our preferred approach [6].

The analytic approach was a two-stage process. First, study-specific odds ratios (ORs) were calculated using unconditional logistic regression models (with low consumption levels as the reference group). The majority of the included case–control studies matched on age and gender only. Although matched methods (e.g., from conditional logistic regression) are robust to matching distortion, unmatched methods like unconditional logistic regression appear to be viable options for loose-matching data, e.g., data matched on a small number of demographic variables like age and gender. After that, the estimates were pooled using a random effects meta-analysis approach to calculate an overall estimate and 95% confidence interval (CI). Adjustments were made for the following potential confounders: gender (male, female), age at study entry (< 45 years, 45–49 years, 50–54 years, 55–59 years, 60–64 years, 65–69 years, 70–74 years, > 75 years), smoking status (never smoker/former smoker/current smoker), and pack years (< 9 years, 9–17 years, 18–30 years, 31–46 years, > 47 years). Interactions between age and total fruit and vegetable consumption, gender and total fruit and vegetable consumption, and smoking status and total fruit and vegetable consumption were tested and showed no significant interactions. Nonetheless, besides the overall analysis, subgroup analyses were performed on the two main study-specific matching factors gender and age, as recommended by the study of Smith-Warner et al. [7]. Small study effects were analyzed by funnel plots. A sensitivity analysis was performed, leaving out the only study conducted in Asia. All statistical analyses were performed using Stata software version 14. A two-sided p value < 0.05 was considered statistically significant.

## Results

**Fig. 1:**
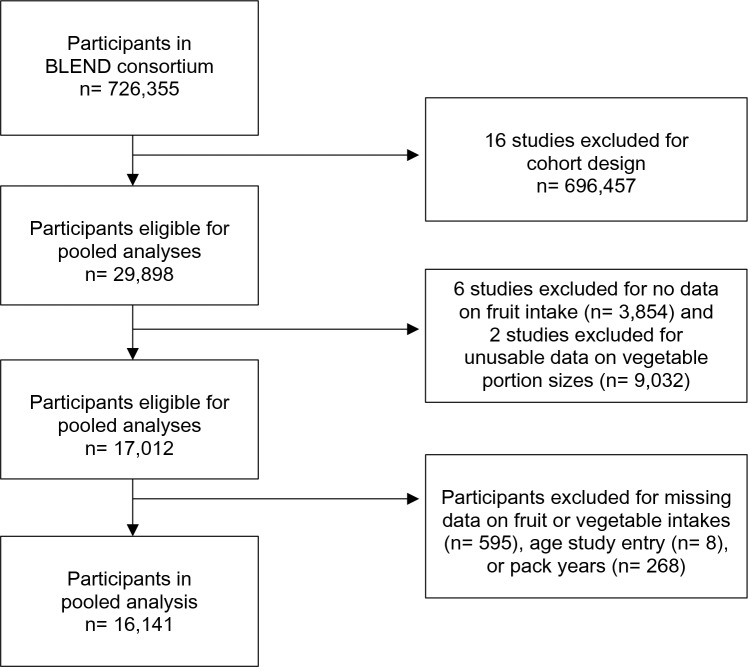
Flow diagram of exclusion criteria for participants included in the pooled case–control analyses on fruit and vegetable intakes

Of the nineteen case–control studies, six studies were excluded for providing no data on fruit or vegetable consumption, and two studies were excluded for providing no data on portion sizes. The eleven included case–control studies [8–18] originated from Belgium, Italy, Sweden, China, Canada, and the USA. In total, out of 17,012 eligible participants, 871 were excluded for having missing data on fruit or vegetable intakes (n = 595), age at study entry (n = 8), or pack years (n = 268) (Fig. 1).

Baseline characteristics of the included studies are presented in Table 2. A total of 5637 BC cases and 10,504 controls were analyzed. Most of these included participants were male (68%) and Caucasian (89%). The mean age for BC was 60 years across all studies. Even though all studies made use of an FFQ to measure usual fruit and vegetable consumption (over the preceding one or two years before study enrollment), the number of fruit and vegetable items described in each questionnaire varied widely.

**Table 2:** Characteristics of the 11 eligible case–control studies according to disease status, gender, age, and smoking status

	Los Angeles BC ca–co study		Roswell Park Cancer Institute		Belgian ca–co study on BC		Stockholm ca–co study		New Hampshire BC study		Italian ca–co study on BC	
Country	USA		USA		Belgium		Sweden		USA		Italy	
Case–control study type	Population based		Population based		Population based		Population based		Population based		Hospital based	
Number of food items asked	49		44		788		188		121		21	
	No.^a^	%	No.^a^	%	No.^a^	%	No.^a^	%	No.^a^	%	No.^a^	%
Total participants	3124	100	862	100	576	100	739	100	567	100	1696	100
Cases	1603	51.31	213	24.71	194	33.68	238	32.21	331	58.38	682	40.21
Controls	1521	48.69	649	75.29	382	66.32	501	67.79	236	41.62	1014	59.79
Men	2451	78.46	650	75.41	394	68.40	415	56.16	380	67.02	1297	76.47
Women	673	21.54	212	24.59	182	31.60	324	43.84	187	32.98	399	23.53
Age												
< 45	374	11.97	46	5.33	3	0.52	0	0	44	7.76	101	5.96
45–49	331	10.60	34	3.94	8	1.39	0	0	30	5.29	117	6.90
50–54	548	17.54	67	7.77	71	12.33	39	5.28	49	8.64	192	11.32
55–59	884	28.30	109	12.65	102	17.71	79	10.69	85	14.99	275	16.21
60–64	887	28.39	153	17.75	110	19.10	114	15.43	85	14.99	355	20.93
65–69	100	3.20	175	20.30	97	16.84	172	23.27	143	25.22	344	20.28
70–74	0	0	162	18.79	77	13.37	263	35.59	131	23.10	224	13.21
> 75	0	0	116	13.46	108	18.75	72	9.74	0	0	88	5.19
Smoking status												
Never smoker	798	25.54	366	42.46	190	32.99	261	35.32	154	27.16	512	30.19
Former smoker	1100	35.21	313	36.31	285	49.48	240	32.48	268	47.27	525	30.40
Current smoker	1226	39.24	183	21.23	101	17.53	238	32.21	145	25.57	672	39.62

	Brescia BC study		NESCC		South and East China ca–co study		Molecular epidemiology of BC		Italian ca–co study	
Country	Italy		Canada		China		USA		Italy	
Case–control study type	Hospital based		Population based		Hospital based		Hospital based		Hospital based	
Number of food items asked	40		69		52		90		77	
	No.^b^	%	No.^b^	%	No.^b^	%	No.^b^	%	No.^b^	%
Total participants	390	100	5580	100	943	100	378	100	1286	100
Cases	185	47.44	884	15.84	479	50.80	180	47.62	648	50.39
Controls	205	52.56	4696	84.16	464	49.20	198	52.38	638	49.61
Men	390	100	2923	52.38	750	79.53	302	79.89	1096	85.23
Women	0	0	2657	47.62	193	20.47	76	20.11	190	14.77
Age										
< 45	29	7.44	970	17.38	91	9.65	108	28.57	47	3.65
45–49	15	3.85	473	8.48	37	3.92	38	10.05	40	3.11
50–54	30	7.69	478	8.57	95	10.07	36	9.52	93	7.23
55–59	48	12.31	568	10.18	118	12.51	36	9.52	132	10.26
60–64	75	19.23	820	14.70	110	11.66	37	9.79	208	16.17
65–69	70	17.95	1082	19.39	121	12.83	62	16.40	295	22.94
70–74	74	18.97	1111	19.91	137	14.53	25	6.61	265	20.61
> 75	49	12.56	78	1.40	234	24.81	36	9.52	206	16.02
Smoking status										
Never smoker	65	16.67	2073	37.15	387	41.04	139	36.77	328	25.51
Former smoker	176	45.13	2353	42.17	228	24.18	194	51.32	562	43.70
Current smoker	149	38.21	1161	20.81	328	34.78	45	11.90	400	31.10

^a^As a result of exclusion criteria, the baseline cohort size and number of cases included in the pooled analyses may differ from original study-specific publications

^b^As a result of exclusion criteria, the baseline case–control size and number of cases included in the pooled analyses may differ from original study-specific publications

### Total fruits

**Fig. 2:**
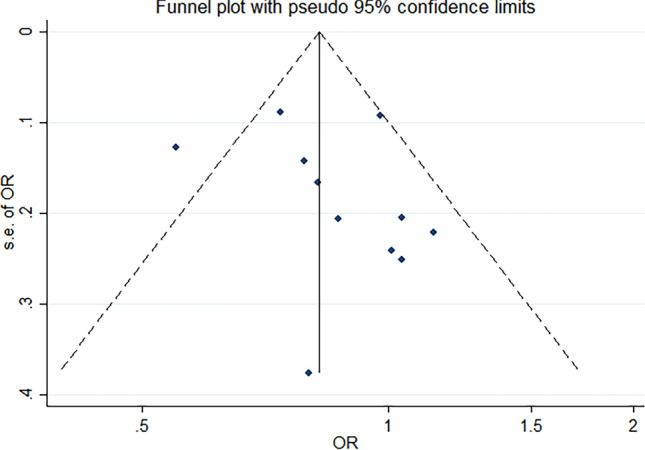
Funnel plot of overall fruit analyses results

Among the eleven included studies, nine demonstrated a trend towards reduced BC risk with higher intake of total fruit, although the association was not always statistically significant (Table 3, Fig. 2). Remarkably, one study reported a non-significant increased risk (Table 3). However, upon pooled analysis, a reduction in BC risk associated with higher total fruit intake was observed. In the overall pooled analysis, greater intakes of fruit showed a reduced BC risk compared to the lowest intakes of fruit (OR 0.79; 95% CI 0.68–0.91) (Table 3). The tests for heterogeneity showed moderate heterogeneity for the association between total fruit and BC (I^2^ = 42.8%).

**Table 3:** Odds ratios of bladder cancer by total fruit intake for all participants

	Los Angeles BC ca–co study	Roswell Park Cancer Institute	Belgian ca–co study on BC	Stockholm ca–co study	New Hampshire BC study	Italian ca–co study on BC	Brescia BC study	NESCC	South and East China ca–co study	Molecular epidemiology of BC	Italian ca–co study	Pooled estimate	Test for heterogeneity
Total fruits													
Q1	Ref	Ref	Ref	Ref	Ref	Ref	Ref	Ref	Ref	Ref	Ref	Ref	
Q2	0.97 (0.81–1.16)	0.79 (0.53–1.18)	0.78 (0.49–1.24)	0.88 (0.59–1.32)	1.15(0.74–1.77)	1.16 (0.94–1.43)	0.84 (0.50–1.41)	0.82 (0.68–0.98)	0.79 (0.57–1.08)	0.85 (0.41–1.76)	1.13 (0.85–1.50)	0.91 (0.83–0.99)	I^2^ = 4.2%, p = 0.403
Q3	0.87 (0.73–1.05)	0.77 (0.51–1.17)	0.51 (0.31–0.83)	0.88 (0.58–1.34)	1.01 (0.65–1.57)	0.87 (0.52–1.47)	1.41 (0.85–2.35)	0.84 (0.69–1.01)	0.56 (0.41–0.78)	0.89 (0.42–1.88)	0.93 (0.70–1.23)	0.79 (0.68–0.91)	I^2^ = 42.8%, p = 0.065
Citrus fruits													
Q1	Ref	Ref	Ref	Ref	Ref		Ref	Ref	Ref	Ref	Ref	Ref	
Q2	0.97 (0.81–1.16)	0.59 (0.40–0.85)	1.04 (0.66–1.66)	1.02 (0.70–1.49)	0.93 (0.60–1.44)		0.81 (0.49–1.34)	0.97 (0.82–1.14)	0.78 (0.54–1.13)	0.59 (0.26–1.33)	0.98 (0.76–1.26)	0.88 (0.78–0.99)	I^2^ = 25.1%, p = 0.213
Q3	0.85 (0.71–1.02)	0.61 (0.39–0.96)	0.72 (0.45–1.16)	0.75 (0.46–1.21)	0.96 (0.62–1.50)		1.06 (0.64–1.77)	1.03 (0.84–1.27)	0.52 (0.38–0.72)	0.46 (0.18–1.14)	1.56 (1.09–2.23)	0.81 (0.65–0.98)	I^2^ = 66.2%, p = 0.002
Soft fruits													
Q1				Ref	Ref		Ref		Ref		Ref	Ref	
Q2				1.12 (0.62–2.03)	0.62 (0.41–0.93)		0.99 (0.59–1.63)		0.71 (0.52–0.96)		0.96 (0.73–1.27)	0.79 (0.63–0.96)	I^2^ = 26.1%, p = 0.247
Q3				1.43 (0.98–2.08)	1.44 (0.87–2.36)		2.08 (1.22–3.55)		0.54 (0.39–0.75)		1.01 (0.75–1.35)	1.14 (0.69–1.60)	I^2^ = 81.5%, p = 0.000
Stone fruits													
Q1		Ref		Ref	Ref		Ref		Ref		Ref	Ref	
Q2		0.64 (0.42–0.97)		0.78 (0.54–1.11)	1.08 (0.70–1.65)		1.67 (1.00–2.80)		1.30 (1.01–1.67)		1.30 (1.01–1.67)	1.05 (0.77–1.33)	I^2^ = 71.2%, p = 0.004
Q3		1.00 (0.68–1.48)		0.71 (0.42–1.19)	0.95 (0.61–1.48)		1.66 (1.00–2.75)		0.46 (0.35–0.62)		1.27 (0.90–1.79)	0.91 (0.58–1.23)	I^2^ = 78.8%, p = 0.000
Pome fruits													
Q1		Ref		Ref	Ref		Ref	Ref		Ref	Ref	Ref	
Q2		0.92 (0.64–1.31)		0.82 (0.55–1.21)	1.32 (0.77–2.28)		1.41 (0.90–2.23)	0.90 (0.76–1.08)		0.54 (0.24–1.21)	0.94 (0.71–1.23)	0.90 (0.74–1.06)	I^2^ = 39.6%, p = 0.128
Q3		0.67 (0.39–1.14)		0.90 (0.59–1.38)	1.03 (0.69–1.54)		0.74 (0.32–1.70)	0.76 (0.62–0.93)		0.45 (0.21–0.97)	0.80 (0.59–1.08)	0.76 (0.65–0.87)	I^2^ = 0.0%, p = 0.517
Tropical fruits													
Q1	Ref	Ref		Ref	Ref		Ref	Ref	Ref	Ref	Ref	Ref	
Q2	1.15 (0.96–1.38)	0.98 (0.66–1.43)		1.00 (0.67–1.49)	0.88 (0.56–1.36)		1.30 (0.77–2.19)	0.96 (0.80–1.14)	0.72 (0.54–0.96)	1.92 (0.71–5.18)	1.15 (0.87–1.53)	0.98 (0.86–1.10)	I^2^ = 26.8%, p = 0.205
Q3	0.97 (0.81–1.16)	0.90 (0.59–1.40)		0.91 (0.61–1.37)	1.06 (0.69–1.64)		0.99 (0.61–1.62)	0.81 (0.67–0.99)	0.56 (0.37–0.83)	1.45 (0.63–3.33)	0.80 (0.60–1.06)	0.84 (0.73–0.94)	I^2^ = 20.5%, p = 0.261

Adjusted for gender, age at study entry (< 45 years, 45–49 years, 50–54 years, 55–59 years, 60–64 years, 65–69 years, 70–74 years, > 75 years), smoking status (current smoker/former smoker/never smoker), and pack years (< 9 years, 9–17 years, 18–30 years, 31–46 years, > 47 years)

In the subgroup analysis on gender, similar results were observed in both men and women, showing a pooled decreased BC risk among those consuming the highest intakes of fruit (OR_men_ = 0.83; 95% CI 0.71–0.95) (Table 4), and OR_women_ = 0.60; 95% CI 0.40–0.80 (Table 5)). Whilst the heterogeneity among men for the might not be considerable (I^2^ = 25.7%), the heterogeneity among women may represent moderate heterogeneity (I^2^ = 43.3%) (Appendix 2, Tables 6, 7).

**Table 4:** Odds ratios of bladder cancer by total vegetable intake for all participants

	Los Angeles BC ca–co study	Roswell Park Cancer Institute	Belgian ca–co study on BC	Stockholm ca–co study	New Hampshire BC study	Italian ca–co study on BC	Brescia BC study	NESCC	South and East China ca–co study	Molecular epidemiology of BC	Italian ca–co study	Pooled estimate	Test for heterogeneity
Total vegetables													
Q1	Ref	Ref	Ref	Ref	Ref	Ref	Ref	Ref	Ref	Ref	Ref	Ref	
Q2	0.95 (0.79–1.13)	0.76 (0.51–1.13)	0.86 (0.54–1.37)	1.12 (0.74–1.68)	1.00 (0.65–1.53)	0.74 (0.57–0.94)	1.05 (0.64–1.72)	1.00 (0.83–1.20)	0.65 (0.47–0.89)	1.30 (0.62–2.69)	0.79 (0.60–1.05)	0.91 (0.83–0.99)	I^2^ = 0.0%, p = 0.442
Q3	0.74 (0.62–0.88)	0.87 (0.58–1.30)	1.01 (0.63–1.62)	1.04 (0.70–1.56)	1.14 (0.74–1.76)	0.55 (0.43–0.71)	1.04 (0.64–1.71)	0.98 (0.82–1.18)	0.82 (0.59–1.13)	0.80 (0.38–1.66)	0.79 (0.60–1.05)	0.82 (0.70–0.94)	I^2^ = 52.9%, p = 0.020
Leaf vegetables													
Q1	Ref	Ref	Ref	Ref	Ref	Ref	Ref	Ref	Ref	Ref	Ref	Ref	
Q2	0.82 (0.69–0.98)	0.68 (0.46–1.00)	0.70 (0.44–1.12)	0.82 (0.56–1.20)	0.94 (0.61–1.45)	0.65 (0.43–0.98)	0.59 (0.36–0.97)	1.16 (0.96–1.39)	1.36 (0.99–1.87)	0.77 (0.36–1.64)	0.81 (0.63–1.05)	0.84 (0.71–0.96)	I^2^ = 50.7%, p = 0.027
Q3	0.76 (0.63–0.91)	0.55 (0.36–0.83)	0.82 (0.51–1.32)	0.75 (0.48–1.17)	0.94 (0.62–1.44)	0.59 (0.47–0.74)	0.97 (0.57–1.66)	1.07 (0.89–1.29)	1.38 (1.00–1.91)	0.82 (0.40–1.69)	0.92 (0.67–1.26)	0.82 (0.68–0.96)	I^2^ = 63.9%, p = 0.002
Brassica													
Q1			Ref		Ref							Ref	
Q2			0.74 (0.47–1.17)		0.85 (0.54–1.36)							0.79 (0.52–1.06)	I^2^ = 0.0%, p = 0.687
Q3			0.82 (0.50–1.35)		1.53 (0.91–2.59)							1.08 (0.40–1.76)	I^2^ = 55.0%, p = 0.136
Stalk vegetables													
Q1		Ref	Ref	Ref	Ref		Ref		Ref		Ref	Ref	
Q2		0.94 (0.19–4.61)	0.92 (0.58–1.47)	1.05 (0.72–1.52)	0.87 (0.57–1.33)		1.18 (0.73–1.90)		0.89 (0.67–1.19)		0.97 (0.71–1.33)	0.95 (0.80–1.09)	I^2^ = 0.0%, p = 0.977
Q3		0.39 (0.07–2.21)	0.97 (0.61–1.54)	1.06 (0.67–1.68)	1.17 (0.73–1.88)		0.77 (0.45–1.33)		1.34 (0.89–2.00)		0.66 (0.50–0.87)	0.87 (0.67–1.08)	I^2^ = 33.5%, p = 0.172
Shoot vegetables													
Q1	Ref	Ref	Ref	Ref	Ref		Ref	Ref	Ref		Ref	Ref	
Q2	1.00 (0.84–1.18)	0.88 (0.60–1.29)	0.92 (0.58–1.46)	1.53 (1.02–2.28)	0.95 (0.64–1.40)		1.06 (0.63–1.76)	0.97 (0.82–1.15)	1.13 (0.85–1.50)		0.88 (0.66–1.15)	0.97 (0.88–1.06)	I^2^ = 0.0%, p = 0.658
Q3	0.85 (0.71–1.01)	0.87 (0.58–1.32)	0.88 (0.55–1.42)	1.16 (0.76–1.77)	1.16 (0.64–2.10)		0.95 (0.57–1.59)	0.86 (0.69–1.06)	0.92 (0.60–1.40)		0.85 (0.64–1.12)	0.87 (0.78–0.96)	I^2^ = 0.0%, p = 0.967
Tubers													
Q1	Ref		Ref	Ref	Ref		Ref					Ref	
Q2	1.04 (0.88–1.24)		0.84 (0.56–1.25)	0.84 (0.56–1.25)	0.86 (0.56–1.34)		0.48 (0.12–1.87)					0.95 (0.81–1.08)	I^2^ = 0.0%, p = 0.534
Q3	0.90 (0.75–1.08)		1.22 (0.79–1.89)	0.86 (0.57–1.30)	0.94 (0.61–1.46)		0.84 (0.22–3.22)					0.92 (0.78–1.06)	I^2^ = 0.0%, p = 0.803

Adjusted for age at study entry (< 45 years, 45–49 years, 50–54 years, 55–59 years, 60–64 years, 65–69 years, 70–74 years, > 75 years), gender, smoking status (current smoker/former smoker/never smoker), and pack years (< 9 years, 9–17 years, 18–30 years, 31–46 years, > 47 years)

Similarly, when analyzing the data according to age (i.e., 60 years and under), consistent results were observed. In the overall pooled analysis, participants younger than 60 years of age with high fruit consumption exhibited a significant decrease in BC risk (OR 0.76; 95% CI 0.64–0.88), with no considerable statistical heterogeneity observed in this group (I^2^ = 0.0%) (Appendix 3, Table 10). Likewise, a decreased BC risk was observed in participants 60 years or older consuming the highest intakes of fruit (OR 0.80; 95% CI 0.69–0.91), with negligible heterogeneity for this association (I^2^ = 5.5%) (Appendix 3, Table 11). Additionally, the sensitivity analysis, excluding the Chinese study [12], yielded similar results compared to the overall analysis (OR 0.83; 95% CI 0.72–0.93).

### Citrus fruits, pome fruits, soft fruits, stone fruits and tropical fruits

No associations between high consumption of soft fruits or stone fruits and BC risk were found (Table 3). Notably, a trend indicating a lower BC risk was observed for soft fruits in four out of the five studies (Table 3), with the exception of the Stockholm case–control study, where an increased BC risk was observed. For citrus fruits, an overall decreased BC risk was noted with high consumption (OR 0.81; 95% CI 0.65–0.98). Interestingly, a similar trend of lower BC risk for citrus fruits was evident in eight out of the ten studies (Table 3), except for the Belgian case–control study on BC and the Stockholm case–control study, which reported an increased BC risk. However, the overall decreased BC risk results for citrus fruit revealed considerable heterogeneity (I^2^ = 66.2). Similar patterns were observed in the stratified analysis by gender and age (i.e., > 60 years and under) (Appendix 2, Tables 6, 7, Appendix 3, Tables 10, 11). Among women participants and participants aged 60 years or more, an overall decreased BC risk was observed with high consumption of citrus fruits (OR 0.56; 95% CI 0.32–0.80, and OR 0.80; 95% CI 0.62–0.99, respectively). However, both analyses showed considerable heterogeneity (I^2^ = 62.5%, and I^2^ = 53.2%, respectively). Conversely, among participants younger than 60 years, an association between the highest intakes of citrus fruits and BC risk was observed (OR 0.77; 95% CI 0.64–0.91) with no considerable statistical heterogeneity (I^2^ = 0.0%). In men, no association was found between greater intakes of citrus fruits and BC risk (Appendix 2, Table 6).

Overall, greater consumption of pome fruits was associated with a decreased BC risk (OR 0.76; 95% CI 0.65–0.87) and statistical heterogeneity was not considerable (I^2^ = 0.0%). In the subgroup analyses, pome fruit consumption (highest *versus* lowest intakes) was associated with a lower BC risk in men (OR 0.77, 95% CI 0.58–0.97), women (OR 0.58, 95% CI 0.42–0.73), < 60 years (OR 0.52, 95% CI 0.31–0.72), and ≥ 60 years (OR 0.85, 95% CI 0.71–0.99), with low to moderate heterogeneity (I^2^ = 40.2%, I^2^ = 0.0%, I^2^ = 15.3%, and I^2^ = 0.0, respectively) (Appendix 2, Tables 6, 7, Appendix 3, Tables 10, 11).

High consumption of tropical fruits was associated with a decreased BC risk in the overall analysis (OR 0.84; 95% CI 0.73–0.94, I^2^ = 20.5%). Again, it should be noted that a trend towards a decreased risk was shown in seven out of the nine studies, while the New Hampshire and Molecular epidemiology of BC studies showed a trend towards an increased BC risk. In the subgroup analyses on age, both age groups (< 60 and ≥ 60 years) showed associations between the highest intakes of tropical fruits and BC risk (OR 0.84; 95% CI 0.70–0.98 and OR 0.83; 95% CI 0.72–0.95, respectively) and no heterogeneity was observed for these associations (Appendix 3, Tables 10 and 11).

### Total vegetables

**Fig. 3:**
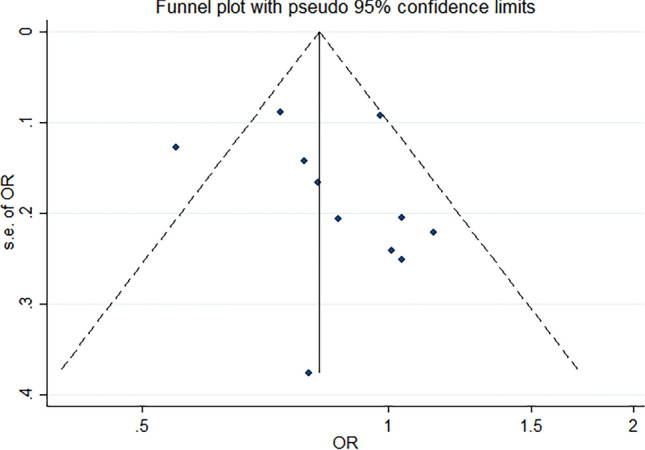
Funnel plot of overall vegetable analyses results

Although high total vegetable consumption was associated with BC risk (OR 0.82; 95% CI 0.70–0.94), heterogeneity was observed (I^2^ = 52.9%) (Table 4, Fig. 3). In men, the highest intakes of total vegetables were associated with a decreased BC risk with no considerable heterogeneity (OR 0.80; 95% CI 0.71–0.88, I^2^ = 1.0%) (Appendix 2, Table 8). Similar results were found for participants ≥ 60 years (OR 0.81; 95% CI 0.71–0.91, I^2^ = 0.0%) (Appendix 3, Table 13). Greater intakes of total vegetables among participants < 60 years were significantly associated with a decreased BC risk (OR 0.70; 95% CI 0.52–0.88). However, substantial heterogeneity was observed for this association (I^2^ = 47.8%) (Appendix 2, Table 3). The sensitivity analysis did not change the result (OR 0.82; 95% CI 0.69–0.95).

### Leaf vegetables, brassica, stalk vegetables, shoot vegetables, and tubers

No associations between high consumptions of brassica, stalk vegetables, and tubers, and BC risk were observed (Table 4). Although subject to substantial heterogeneity, associations were found for the overall high intake of leaf vegetables and decreased BC risk (OR 0.82; 95% CI 0.68–0.96). This trend was observed for nine out of the eleven studies, while the South and East China ca–co study and NESCC study showed a trend towards an increased BC risk (Table 4).

High intakes of shoot vegetables significantly decreased the BC risk with no observed substantial heterogeneity (OR 0.87; 95% 0.78–0.96, I^2^ = 0.0%) (Table 4). In the subgroup analyses, significant associations for leaf vegetables (OR 0.70; 95% CI 0.56–0.84) and shoot vegetables (OR 0.83; 95% CI 0.68–0.97) were found in participants < 60 years with no substantial heterogeneity (I^2^ = 20.0%, and I^2^ = 0.0%, respectively) (Appendix 3, Table 12). Although associations were found for leaf vegetables and BC risk in men, women, and participants ≥ 60 years, substantial heterogeneity was observed for these associations (Appendix 2, Tables 8, 9, Appendix 3, Table 13).

## Discussion

In this large pooled analysis of eleven case–control studies, comprising 5637 cases and 10,504 controls, significant inverse associations were found between high fruit and vegetable consumption and BC risk. There were inverse associations between total fruit, citrus fruit, pome fruit, and tropical fruit consumption and BC risk. No associations were found for high consumption of soft fruits or stone fruits. For vegetable consumption, inverse associations were found between total vegetable, leaf vegetable, and shoot vegetable consumption and a BC risk. Brassica, stalk vegetables, and tubers were not associated with BC risk.

It seems plausible that substances in fruits and vegetables including minerals, phytochemicals, and antioxidant nutrients, have potentially anticarcinogenic properties to protect against the development of cancer [1, 19]. Throughout Europe and the USA, apples and pears are the most consumed pome fruits. Apples contain a wide range of phytochemicals which have been found to have strong antioxidant properties and the ability to inhibit cancer cell proliferation [20]. Hence, results from an Italian case–control study and from the EPIC study, which included data from ten European countries, confirm that greater consumption of apples and pears decreases the BC risk (OR 0.63; 95% CI 0.39–0.99, and OR 0.90; 95% CI 0.82–0.98, respectively) [21, 22]. In addition, scientific evidence suggests that vitamin C, an essential nutrient abundant in citrus fruits, exerts anticancer effects in the bladder through diverse pathways. These include a malignancy-inhibiting shift in the transcriptome, as well as elevating levels of 5-hydroxymethylcytosine [23].

Brassica vegetables also contain high levels of phytochemicals, such as glucosinolates and isothiocyanates, and are therefore expected to lower the BC risk [19, 24]. This, however, was not confirmed in this study. Leafy vegetables contain high concentrations of carotenoids that could potentially protect against the damage to DNA by scouring free radicals [25]. Hence, results of a meta-analysis indicated that per 0.2 serving increment of daily green leafy vegetable consumption, the BC risk decreases with 2% [26]. We observed evidence of an inverse association between consumption of leafy vegetables and BC risk, which is consistent with these potential biological mechanisms.

Although no previous studies investigated specifically the role of shoot vegetables or tropical fruits in BC risk, these vegetables and fruits contain a wide range of carotenoids, folates, vitamins, and carotene, which may offer protection against the development of BC [27].

In line with our research a meta-analysis conducted in East Asians showed an inverse association between total fruit intake and BC risk [23]. Furthermore, within our BLEND study investigating fruit intake and BC risk among thirteen cohort studies, an inverse association between fruit intake and BC risk was found in women, but not in men. Nor was an association found for any fruit subgroup [28]. These null associations were confirmed in a Japanese cohort study including 1,287,514 person-years of follow-up. In addition, a null association was also found for total vegetable intake and any subgroup vegetable [29]. These observed differences might be due to the difference in study design. Although both case–control and cohort studies are subject to (a different form of) selection bias, and other methodological limitations, such as measurement error, there is an important methodological difference between these different study types. While in case–control studies, the assessment of lifestyle occurs after diagnosis, in cohort studies, the assessment of lifestyle occurs prior to diagnosis. It is therefore thought that case–control studies are more prone to recall bias and provide a lower certainty of evidence than cohort studies [30, 31]. However, since some of our results have low heterogeneity, the role of bias is likely to have minimal influence on these results [32]. In addition, recall bias has been addressed and analyzed for its consequences in many epidemiological/methodological papers, and no clear answer on the magnitude of the effect of this specific type of bias could be drawn.

Several of our results revealed discrepancies among individual study findings and indicated significant heterogeneity in the effects of fruit and vegetable consumption on BC risk. This aligns with previous research highlighting the challenges of comparing diet-disease relationships globally due to variations in dietary habits and assessment methods across populations. For instance, differences in portion size estimations, nutrient databases used, and other factors contribute to result heterogeneity, complicating generalization and interpretation. Therefore, while our analysis focused on individual food items rather than overall dietary patterns, it is essential to consider the broader context of dietary diversity and assessment methods when interpreting these findings.

## Strengths and limitations

This study has some limitations. First, the number of fruit and vegetable items described in each FFQ varied widely across the studies. Although it has been reported that fruit and vegetable servings increase with the number of fruit and vegetable items on a questionnaire [33], the total of fruit and vegetable questions on the FFQs did not significantly modify the association between fruit or vegetable consumption and BC risk. In addition, the number of studies included in the fruit and vegetable subgroup analyses varied depending on whether the items comprising a particular fruit or vegetable subgroup were asked on the study-specific FFQs. Consequently, the power to examine associations for some subgroups is more limited compared with that for analyses of total fruit and total vegetables. Second, while case–control studies are valuable in investigating associations, they inherently possess limitations, particularly when assessing dietary factors which may be influenced by participants' cancer status [34]. Furthermore, as mentioned previously, case–control studies are generally considered to have higher risk of recall bias than cohort studies as the selection of the controls may be subject to population stratification [30]. We acknowledge that cohort studies and randomized controlled trials generally provide stronger evidence. Despite this, our analysis accounts for potential biases by addressing low heterogeneity across studies, suggesting minimal impact on our findings [32]. At last, while limited data on other potential BC risk factors such as body mass index and socioeconomic status were available, current literature suggests their contribution to BC risk is relatively small [35–38]. Besides, this pooled analysis also has several strengths, including the large sample size with harmonized variables across multiple studies, providing high statistical power to examine the role of total fruits and vegetables, subgroups of fruits and vegetables, and the possibility to perform subgroup analyses on gender and age. Moreover, we found low heterogeneity between studies for many of the association reported, the role of bias is likely to have minimal influence on our results.

## Conclusion

This comprehensive study provides compelling evidence that the consumption of fruits overall, citrus fruits, pome fruits and tropical fruits reduce the BC risk. Besides, evidence was found for an inverse association between total vegetables and shoot vegetables intake.

## Data Availability

The data that support the findings of this study are available on request from the corresponding author.

## Appendix 1: Food frequency questionnaires (Table 5)

**Table 5:** Detailed information on FFQs

Study name	Summary of dietary collection method	Food items asked (n)
The Los Angeles Bladder Cancer Study [39]	In-person interviews were conducted at subjects' homes, collecting information up to 2 years prior to cancer diagnosis for cases and index case diagnosis for controls. The structured questionnaire included forty food groups/items to assess dietary intake of vitamins A and C, carotenoids	49
South and East China case–control study on bladder and prostate cancer [12]	Trained interviewers, aware of the case–control status, used a food frequency questionnaire to collect data on the consumption of 35 specific food items within eight food groups, including dairy, eggs, fish, fruit, grain, meat, soy, and vegetables. Dietary information reflected the period of 1 year before the interview, with food intake categorized into six levels ranging from no intake to daily consumption	52
Belgian Case–Control study on Bladder Cancer [14]	The FFQ used was derived from the IMMIDIET study and comprised 788 food items, drawn from three existing food tables. Participants estimated their usual diet up to 12 months prior to the interview, reporting frequency and quantity of intake for each item	788
New Hampshire Bladder Cancer study [27]	A 121-item semi-quantitative FFQ, developed by Willett et al. for the Nurses' Health Study [REF], was used to assess participants' usual dietary intake. The questionnaire included food groups such as dairy, fruit, vegetables, eggs and meat, breads, beverages, and baked goods	121
Italian Case–Control study on Bladder Cancer [40]	Trained interviewers used a comprehensive standardized questionnaire to gather information from case patients and control subjects, covering sociodemographic details, lifestyle habits, medical history, occupational exposure, and chemical exposure. Interviews were conducted within a year of hospital admission	21
Molecular Epidemiology of Bladder Cancer and Prostate Cancer [9]	Participants were interviewed by a nurse using a standardized questionnaire to collect dietary data, including demographic information and personal habits such as smoking and alcohol consumption	90
The national enhanced cancer surveillance system [41]	A 70-item food frequency questionnaire, along with supplementary dietary habits, was utilized to gather data on the consumption of essential food items and nutrients, spanning two and 20 years before the interview. Questionnaires were dispatched to the patients within 1–4 months of diagnosis, and at requitement for controls	69
Brescia Bladder Cancer Case–control Study [17]	The dietary data collection process involved face-to-face interviews conducted during hospitalization by three experienced interviewers. A comprehensive semistructured questionnaire covered demographic, dietary, environmental, and occupational factors. It included sections on sociodemographic status, clinical data, dietary habits, tobacco and alcohol consumption, fluid intake, environmental exposure, and occupational history	40
Roswell Park Cancer Institute [18]	A 44-item food frequency questionnaire designed to assess dietary habits several years before diagnosis, focusing on intake of fruits, vegetables, cruciferous vegetables, and foods rich in vitamins A, C, and E, fat, and fiber was used	44
Stockholm case–control study [42]	Diet was assessed by an extensive semiquantitative food-frequency questionnaire including 188 food items (27 fried meat dishes, 16 roasted meat dishes, 11 boiled meat dishes, four grilled meat dishes, four gravies and sauces, seven fish dishes, fried eggs, omelettes, and black pudding). All questions referred to eating habits 5 years previously	188
Italian case–control study [43]	Trained personnel administered a standard, structured questionnaire to study subjects. Patients’ dietary habits in the 2 years preceding study enrolment were collected through a validated and reproducable FFQ	77

*FFQ* food frequency questionnaire, *BC* bladder cancer

## Appendix 2: Gender stratified results (Tables 6, 7, 8, 9)

**Table 6:** Odds ratios of bladder cancer by total fruit intake among men

	Los Angeles BC ca–co study	Roswell Park Cancer Institute	Belgian ca–co study on BC	Stockholm ca–co study	New Hampshire BC study	Italian ca–co study on BC	Brescia BC study	NESCC	South and East China ca–co study	Molecular epidemiology of BC	Italian ca–co study	Pooled estimate	Test for heterogeneity
Total fruits													
Q1	Ref	Ref	Ref	Ref	Ref	Ref	Ref	Ref	Ref	Ref	Ref	Ref	
Q2	1.04 (0.86–1.27)	0.86 (0.55–1.35)	0.81 (0.48–1.36)	0.81 (0.51–1.31)	1.08 (0.63–1.84)	1.26 (1.00–1.60)	0.84 (0.50–1.41)	0.81 (0.64–1.01)	0.78 (0.55–1.10)	0.69 (0.32–1.51)	1.19 (0.87–1.62)	0.92 (0.81–1.02)	I^2^ = 18.8%, p = 0.264
Q3	0.94 (0.76–1.15)	0.80 (0.49–1.31)	0.47 (0.27–0.83)	0.88 (0.52–1.46)	1.02 (0.60–1.74)	0.93 (0.51–1.69)	1.41 (0.85–2.35)	0.93 (0.74–1.17)	0.71 (0.49–1.02)	0.71 (0.31–1.63)	0.88 (0.64–1.19)	0.83 (0.71–0.95)	I^2^ = 25.7%, p = 0.199
Citrus fruits													
Q1	Ref	Ref	Ref	Ref	Ref		Ref	Ref	Ref	Ref	Ref	Ref	
Q2	1.05 (0.86–1.28)	0.61 (0.40–0.93)	1.05 (0.63–1.77)	1.14 (0.74–1.77)	0.96 (0.56–1.65)		0.81 (0.49–1.34)	0.94 (0.76–1.17)	0.79 (0.52–1.19)	0.69 (0.29–1.65)	0.95 (0.73–1.26)	0.86 (0.73–1.00)	I^2^ = 40.9%, p = 0.085
Q3	0.93 (0.75–1.14)	0.76 (0.45–1.30)	0.79 (0.46–1.37)	0.68 (0.37–1.26)	0.87 (0.51–1.48)		1.06 (0.64–1.77)	1.07 (0.82–1.39)	0.65 (0.45–0.93)	0.49 (0.18–1.32)	1.57 (1.07–2.32)	0.87 (0.72–1.01)	I^2^ = 32.5%, p = 0.148
Pome fruits													
Q1		Ref		Ref	Ref		Ref	Ref		Ref	Ref	Ref	
Q2		0.98 (0.65–1.49)		0.87 (0.55–1.40)	1.34 (0.69–2.60)		1.41 (0.90–2.23)	0.97 (0.78–1.21)		0.45 (0.18–1.12)	1.00 (0.75–1.34)	0.94 (0.78–1.11)	I^2^ = 22.5%, p = 0.258
Q3		0.77 (0.41–1.45)		0.79 (0.47–1.34)	1.22 (0.74–2.02)		0.74 (0.32–1.70)	0.83 (0.65–1.07)		0.36 (0.15–0.85)	0.94 (0.67–1.31)	0.77 (0.58–0.97)	I^2^ = 40.2%, p = 0.123
Tropical fruits													
Q1	Ref	Ref		Ref	Ref		Ref	Ref	Ref	Ref	Ref	Ref	
Q2	1.16 (0.95–1.41)	0.82 (0.53–1.28)		0.86 (0.53–1.39)	1.00 (0.58–1.71)		1.30 (0.77–2.19)	1.02 (0.82–1.27)	0.76 (0.55–1.04)	1.67 (0.57–4.92)	1.19 (0.87–1.61)	0.99 (0.88–1.11)	I^2^ = 6.1%, P = 0.384
Q3	0.97 (0.79–1.19)	0.86 (0.52–1.42)		0.85 (0.52–1.40)	1.30 (0.76–2.22)		0.99 (0.61–1.62)	0.88 (0.69–1.12)	0.76 (0.48–1.20)	1.54 (0.61–3.87)	0.78 (0.57–1.07)	0.89 (0.79–1.00)	I^2^ = 0.0%, p = 0.863

Adjusted for age at study entry (< 45 years, 45–49 years, 50–54 years, 55–59 years, 60–64 years, 65–69 years, 70–74 years, > 75 years), smoking status (current smoker/former smoker/never smoker), and pack years (< 9 years, 9–17 years, 18–30 years, 31–46 years, > 47 years)

**Table 7:** Odds ratios of bladder cancer by total fruit intake among women

	Los Angeles BC ca–co study	Roswell Park Cancer Institute	Belgian ca–co study on BC	Stockholm ca–co study	New Hampshire BC study	Italian ca–co study on BC	NESCC	South and East China ca–co study	Molecular epidemiology of BC	Italian ca–co study	Pooled estimate	Test for heterogeneity
Total fruits												
Q1	Ref	Ref	Ref	Ref	Ref	Ref	Ref	Ref	Ref	Ref	Ref	
Q2	0.68 (0.44–1.04)	0.53 (0.22–1.27)	0.68 (0.24–1.97)	1.07 (0.50–2.28)	1.24 (0.58–2.65)	0.81 (0.50–1.31)	0.81 (0.60–1.10)	0.78 (0.36–1.70)	5.68 (0.43–74.39)	0.88 (0.41–1.86)	0.76 (0.61–0.90)	I^2^ = 0.0%, p = 0.781
Q3	0.65 (0.43–0.97)	0.66 (0.29–1.50)	0.60 (0.21–1.67)	1.00 (0.47–2.12)	0.91 (0.42–1.99)	0.70 (0.24–2.09	0.70 (0.51–0.96)	0.25 (0.12–0.53)	2.23 (0.29–17.20)	1.28 (0.59–2.78)	0.60 (0.40–0.80)	I^2^ = 43.3%, p = 0.070
Citrus fruits												
Q1	Ref	Ref	Ref	Ref	Ref		Ref	Ref	Ref	Ref	Ref	
Q2	0.69 (0.46–1.05)	0.51 (0.23–1.13)	0.95 (0.33–2.71)	0.77 (0.38–1.58)	0.96 (0.45–2.05)		1.02 (0.77–1.35)	0.79 (0.34–1.84)	0.04 (0.00–2.51)	1.14 (0.60–2.17)	0.78 (0.61–0.95)	I^2^ = 6.6%, p = 0.380
Q3	0.62 (0.42–0.92)	0.39 (0.17–0.92)	0.52 (0.19–1.45)	0.82 (0.38–1.80)	1.26 (0.57–2.75)		0.99 (0.70–1.41)	0.26 (0.13–0.51)	0.03 (0.00–1.45)	1.47 (0.56–3.85)	0.56 (0.32–0.80)	I^2^ = 62.5%, p = 0.006
Pome fruits												
Q1		Ref		Ref	Ref		Ref		Ref	Ref	Ref	
Q2		0.76 (0.37–1.59)		0.74 (0.35–1.57)	1.37 (0.53–3.59)		0.82 (0.61–1.09)		2.33 (0.25–21.73)	0.62 (0.30–1.29)	0.78 (0.59–0.97)	I^2^ = 0.0%, p = 0.953
Q3		0.45 (0.16–1.24)		1.21 (0.59–2.49)	0.68 (0.34–1.36)		0.66 (0.47–0.92)		1.81 (0.22–14.98)	0.33 (0.15–0.74)	0.58 (0.42–0.73)	I^2^ = 0.0%, p = 0.476
Tropical fruits												
Q1	Ref	Ref		Ref	Ref		Ref	Ref	Ref	Ref	Ref	
Q2	1.18 (0.76–1.81)	1.58 (0.71–3.52)		1.44 (0.68–3.07)	0.64 (0.29–1.38)		0.86 (0.64–1.15)	0.52 (0.25–1.06)	2.85 (0.20–40.70)	0.94 (0.43–2.05	0.82 (0.63–1.01)	I^2^ = 6.2%, p = 0.383
Q3	0.99 (0.66–1.48)	1.10 (0.44–2.75)		1.21 (0.57–2.57)	0.67 (0.31–1.44)		0.70 (0.51–0.95)	0.19 (0.08–0.47)	1.04 (0.12–9.21)	0.83 (0.39–1.78)	0.69 (0.39–1.00)	I^2^ = 69.6%, p = 0.002

Adjusted for age at study entry (< 45 years, 45–49 years, 50–54 years, 55–59 years, 60–64 years, 65–69 years, 70–74 years, > 75 years), smoking status (current smoker/former smoker/never smoker), and pack years (< 9 years, 9–17 years, 18–30 years, 31–46 years, > 47 years)

**Table 8:** Odds ratios of bladder cancer by total vegetable intake stratified by men

	Los Angeles BC ca–co study	Roswell Park Cancer Institute	Belgian ca–co study on BC	Stockholm ca–co study	New Hampshire BC study	Italian ca–co study on BC	Brescia BC study	NESCC	South and East China ca–co study	Molecular epidemiology of BC	Italian ca–co study	Pooled estimate	Test for heterogeneity
Total vegetables													
Q1	Ref	Ref	Ref	Ref	Ref	Ref	Ref	Ref	Ref	Ref	Ref	Ref	
Q2	0.90 (0.74–1.11)	0.92 (0.58–1.45)	0.72 (0.43–1.22)	1.25 (0.76–2.05)	0.85 (0.50–1.44)	0.72 (0.55–0.95)	1.01 (0.61–1.68)	0.99 (0.79–1.25)	0.56 (0.40–0.81)	0.86 (0.39–1.92)	0.68 (0.50–0.93)	0.80 (0.70–0.91)	I^2^ = 26.8%, p = 0.189
Q3	0.71 (0.58–0.86)	1.29 (0.80–2.06)	0.93 (0.55–1.60)	0.90 (0.55–1.45)	0.94 (0.55–1.62)	0.69 (0.52–0.91)	1.11 (0.67–1.86)	0.97 (0.77–1.22)	0.91 (0.63–1.32)	0.62 (0.28–1.41)	0.77 (0.56–1.04)	0.80 (0.71–0.88)	I^2^ = 1.0%, p = 0.432
Leaf vegetables													
Q1	Ref	Ref	Ref	Ref	Ref	Ref	Ref	Ref	Ref	Ref	Ref	Ref	
Q2	0.80 (0.66–0.97)	0.81 (0.52–1.26)	0.65 (0.39–1.11)	0.80 (0.50–1.26)	1.23 (0.72–2.10)	0.57 (0.36–0.90)	0.59 (0.36–0.97)	1.27 (1.00–1.60)	1.62 (1.13–2.32)	0.72 (0.32–1.65)	0.77 (0.59–1.02)	0.84 (0.68–0.99)	I^2^ = 57.5%, p = 0.009
Q3	0.76 (0.62–0.93)	0.70 (0.44–1.13)	0.62 (0.36–1.07)	0.70 (0.41–1.19)	0.86 (0.51–1.45)	0.71 (0.55–0.92)	0.97 (0.57–1.66)	1.14 (0.90–1.45)	1.72 (1.19–2.48)	0.61 (0.28–1.35)	1.03 (0.72–1.45)	0.84 (0.70–0.98)	I^2^ = 46.1%, p = 0.046
Shoot vegetables													
Q1	Ref	Ref	Ref	Ref	Ref		Ref	Ref	Ref		Ref	Ref	
Q2	1.09 (0.90–1.33)	1.13 (0.72–1.75)	0.89 (0.53–1.49)	1.53 (0.93–2.53)	1.14 (0.70–1.85)		1.06 (0.63–1.76)	0.90 (0.73–1.11)	1.26 (0.92–1.73)		0.79 (0.59–1.07)	0.99 (0.88–1.11)	I^2^ = 6.8%, p = 0.378
Q3	0.85 (0.70–1.05)	1.24 (0.76–2.01)	0.76 (0.44–1.32)	1.08 (0.65–1.81)	1.49 (0.71–3.15)		0.95 (0.57–1.59)	0.84 (0.64–1.11)	1.34 (0.82–2.17)		0.84 (0.62–1.15)	0.89 (0.78–1.00)	I^2^ = 0.0%, p = 0.751

Adjusted for age at study entry (< 45 years, 45–49 years, 50–54 years, 55–59 years, 60–64 years, 65–69 years, 70–74 years, > 75 years), smoking status (current smoker/former smoker/never smoker), and pack years (< 9 years, 9–17 years, 18–30 years, 31–46 years, > 47 years)

**Table 9:** Odds ratios of bladder cancer by total vegetable intake stratified by women

	Los Angeles BC ca–co study	Roswell Park Cancer Institute	Belgian ca–co study on BC	Stockholm ca–co study	New Hampshire BC study	Italian ca–co study on BC	NESCC	South and East China ca–co study	Molecular epidemiology of BC	Italian ca–co study	Pooled estimate	Test for heterogeneity
Total vegetables												
Q1	Ref	Ref	Ref	Ref	Ref	Ref	Ref	Ref	Ref	Ref	Ref	
Q2	1.21 (0.82–1.79)	0.40 (0.17–0.93)	0.82 (0.28–2.39)	0.90 (0.43–1.91)	1.41 (0.65–3.10)	0.62 (0.35–1.10)	1.05 (0.77–1.43)	1.16 (0.54–2.52)	2.97 (0.38–23.27)	1.01 (0.45–2.23)	0.86 (0.64–1.08)	I^2^ = 24.1%, p = 0.222
Q3	0.89 (0.59–1.33)	0.38 (0.18–0.84)	0.87 (0.32–2.39)	1.24 (0.59–2.64)	1.59 (0.74–3.44)	0.18 (0.09–0.34)	0.98 (0.72–1.34)	0.65 (0.33–1.31)	2.50 (0.32–19.57)	0.67 (0.32–1.44)	0.71 (0.39–1.02)	I^2^ = 78.2%, p = 0.000
Leaf vegetables												
Q1	Ref	Ref	Ref	Ref	Ref	Ref	Ref	Ref	Ref	Ref	Ref	
Q2	1.01 (0.68–1.49)	0.42 (0.19–0.94)	0.64 (0.20–2.04)	0.86 (0.43–1.72)	0.53 (0.24–1.18)	0.85 (0.35–2.08)	1.00 (0.73–1.37)	0.69 (0.35–1.36)	1.60 (0.21–12.15)	0.85 (0.42–1.73)	0.77 (0.61–0.94)	I^2^ = 0.0%, p = 0.534
Q3	0.77 (0.52–1.15)	0.26 (0.11–0.60)	1.78 (0.62–5.09)	0.96 (0.42–2.20)	1.03 (0.49–2.17)	0.26 (0.15–0.46)	0.95 (0.70–1.28)	0.61 (0.30–1.25)	2.09 (0.31–14.30)	0.51 (0.23–1.10)	0.61 (0.37–0.85)	I^2^ = 68.8%, p = 0.001
Shoot vegetables												
Q1	Ref	Ref	Ref	Ref	Ref		Ref	Ref		Ref	Ref	
Q2	0.71 (0.48–1.04)	0.46 (0.20–1.05)	1.11 (0.35–3.51)	1.48 (0.73–2.98)	0.63 (0.31–1.27)		1.12 (0.85–1.47)	0.70 (0.37–1.31)		1.06 (0.50–2.23)	0.80 (0.60–1.00)	I^2^ = 25.7%, p = 0.224
Q3	0.83 (0.56–1.24)	0.34 (0.15–0.76)	1.46 (0.52–4.14)	1.34 (0.63–2.83)	0.68 (0.25–1.89)		0.89 (0.62–1.28)	0.26 (0.10–0.65)		0.76 (0.36–1.60)	0.65 (0.39–0.90)	I^2^ = 57.2%, p = 0.022

Adjusted for age at study entry (< 45 years, 45–49 years, 50–54 years, 55–59 years, 60–64 years, 65–69 years, 70–74 years, > 75 years), smoking status (current smoker/former smoker/never smoker), and pack years (< 9 years, 9–17 years, 18–30 years, 31–46 years, > 47 years)

## Appendix 3: Age stratified results (Tables 10, 11, 12, 13)

**Table 10:** Odds ratios of bladder cancer by total fruit intake stratified by age < 60 years old

	Los Angeles BC ca–co study	Roswell Park Cancer Institute	Belgian ca–co study on BC	Stockholm ca–co study	New Hampshire BC study	Italian ca–co study on BC	Brescia BC study	NESCC	South and East China ca–co study	Molecular epidemiology of BC	Italian ca–co study	Pooled estimate	Test for heterogeneity
Total fruits													
Q1	Ref	Ref	Ref	Ref	Ref	Ref	Ref	Ref	Ref	Ref	Ref	Ref	
Q2	0.95 (0.77–1.17)	0.75 (0.38–1.47)	0.55 (0.23–1.32)	0.85 (0.27–2.71)	1.35 (0.68–2.68)	0.98 (0.69–1.39)	0.86 (0.35–2.12)	0.83 (0.60–1.13)	0.67 (0.39–1.17)	1.12 (0.48–2.63)	1.45 (0.81–2.62)	0.88 (0.76–1.01)	I^2^ = 0.0%, p = 0.796
Q3	0.79 (0.63–0.98)	0.80 (0.39–1.63)	0.44 (0.16–1.18)	1.20 (0.38–3.78)	1.13 (0.52–2.44)	0.83 (0.35–1.96)	1.45 (0.57–3.67)	0.75 (0.53–1.05)	0.58 (0.34–0.99)	0.81 (0.33–1.99)	1.14 (0.63–2.06)	0.76 (0.64–0.88)	I^2^ = 0.0%, p = 0.855
Citrus fruits													
Q1	Ref	Ref	Ref	Ref	Ref	Ref		Ref	Ref	Ref	Ref	Ref	
Q2	0.95 (0.77–1.18)	0.40 (0.21–0.77)	0.84 (0.35–1.98)	0.76 (0.28–2.08)	1.30 (0.63–2.67)	0.84 (0.34–2.11)		1.12 (0.84–1.50)	0.93 (0.49–1.77)	0.36 (0.13–0.99)	1.27 (0.74–2.17)	0.82 (0.59–1.06)	I^2^ = 55.3%, p = 0.017
Q3	0.73 (0.59–0.92)	0.70 (0.32–1.53)	0.86 (0.33–2.24)	1.06 (0.22–5.21)	1.26 (0.58–2.72)	1.09 (0.44–2.70)		1.20 (0.81–1.78)	0.56 (0.33–0.93)	0.65 (0.21–2.06)	1.13 (0.56–2.28)	0.77 (0.64–0.91)	I^2^ = 0.0%, p = 0.743
Pome fruits													
Q1		Ref		Ref	Ref			Ref		Ref	Ref	Ref	
Q2		0.71 (0.39–1.31)		0.57 (0.17–1.87)	1.62 (0.63–4.15)			0.69 (0.50–0.95)		0.49 (0.18–1.32)	1.20 (0.69–2.09)	0.71 (0.53–0.89)	I^2^ = 0.0%, p = 0.600
Q3		0.34 (0.11–1.07)		1.36 (0.40–4.58)	1.33 (0.66–2.66)			0.51 (0.34–0.76)		0.33 (0.13–0.82)	1.07 (0.53–2.17)	0.52 (0.31–0.72)	I^2^ = 15.3%, p = 0.315
Tropical fruits													
Q1	Ref	Ref		Ref	Ref		Ref	Ref	Ref	Ref	Ref	Ref	
Q2	1.03 (0.83–1.27)	1.11 (0.59–2.06)		1.37 (0.42–4.41)	0.94 (0.45–1.96)		0.63 (0.24–1.67)	0.92 (0.68–1.25)	0.74 (0.45–1.21)	1.04 (0.32–3.46)	1.24 (0.68–2.29)	0.95 (0.81–1.10)	I^2^ = 0.0%, p = 0.911
Q3	0.94 (0.76–1.17)	0.65 (0.27–1.53)		1.19 (0.37–3.84)	1.28 (0.61–2.72)		1.29 (0.53–3.13)	0.84 (0.59–1.20)	0.57 (0.30–1.09)	1.05 (0.38–1.09)	0.66 (0.36–1.21)	0.84 (0.70–0.98)	I^2^ = 0.0%, p = 0.703

Adjusted for age at study entry (< 45 years, 45–49 years, 50–54 years, 55–59 years), gender, smoking status (current smoker/former smoker/never smoker), and pack years (< 9 years, 9–17 years, 18–30 years, 31–46 years, > 47 years)

**Table 11:** Odds ratios of bladder cancer by total fruit intake stratified by age ≥ 60 years old

	Los Angeles BC ca–co study	Roswell Park Cancer Institute	Belgian ca–co study on BC	Stockholm ca–co study	New Hampshire BC study	Italian ca–co study on BC	Brescia BC study	NESCC	South and East China ca–co study	Molecular epidemiology of BC	Italian ca–co study	Pooled estimate	Test for heterogeneity
Total fruits													
Q1	Ref	Ref	Ref	Ref	Ref	Ref	Ref	Ref	Ref	Ref	Ref	Ref	
Q2	0.95 (0.68–1.32)	0.76 (0.47–1.25)	0.87 (0.50–1.49)	0.90 (0.59–1.38)	0.94 (0.53–1.68)	1.24 (0.95–1.62)	0.86 (0.46–1.60)	0.85 (0.68–1.06)	0.86 (0.58–1.27)	0.36 (0.08–1.58)	1.07 (0.77–1.48)	0.91 (0.81–1.02)	I^2^ = 0.0%, p = 0.647
Q3	0.93 (0.67–1.28)	0.71 (0.42–1.17)	0.51 (0.29–0.90)	0.84 (0.53–1.31)	0.88 (0.51–1.52)	0.90 (0.47–1.74)	1.40 (0.76–2.58)	0.91 (0.73–1.14)	0.55 (0.37–0.83)	0.66 (0.20–2.18)	0.89 (0.64–1.24)	0.80 (0.69–0.91)	I^2^ = 5.5%, p = 0.391
Citrus fruits													
Q1	Ref	Ref	Ref	Ref	Ref		Ref	Ref	Ref	Ref	Ref	Ref	
Q2	0.99 (0.71–1.37)	0.74 (0.47–1.16)	1.09 (0.63–1.88)	1.08 (0.72–1.61)	0.74 (0.42–1.29)		0.80 (0.44–1.46)	0.92 (0.75–1.13)	0.71 (0.45–1.11)	0.69 (0.19–2.43)	0.93 (0.70–1.23)	0.88 (0.78–0.99)	I^2^ = 0.0%, p = 0.898
Q3	1.03 (0.75–1.42)	0.60 (0.34–1.03)	0.64 (0.37–1.10)	0.77 (0.46–1.26)	0.81 (0.47–1.40)		1.06 (0.57–1.96)	0.99 (0.78–1.27)	0.51 (0.34–0.78)	0.09 (0.01–0.85)	1.74 (1.14–2.66)	0.80 (0.62–0.99)	I^2^ = 53.2%, p = 0.023
Pome fruits													
Q1		Ref		Ref	Ref		Ref	Ref		Ref	Ref	Ref	
Q2		1.04 (0.67–1.62)		0.85 (0.55–1.29)	1.20 (0.61–2.38)		1.53 (0.89–2.64)	1.04 (0.84–1.28)		0.66 (0.17–2.51)	0.90 (0.66–1.23)	0.99 (0.84–1.13)	I^2^ = 0.0%, p = 0.789
Q3		0.85 (0.47–1.54)		0.87 (0.55–1.36)	0.83 (0.50–1.37)		0.92 (0.38–2.25)	0.90 (0.71–1.14)		0.60 (0.17–2.16)	0.78 (0.56–1.10)	0.85 (0.71–0.99)	I^2^ = 0.0%, p = 0.996
Tropical fruits													
Q1	Ref	Ref		Ref	Ref		Ref	Ref	Ref	Ref	Ref	Ref	
Q2	1.40 (1.00–1.94)	0.89 (0.55–1.44)		0.99 (0.64–1.53)	0.82 (0.47–1.44)		1.71 (0.91–3.22)	1.00 (0.80–1.23)	0.70 (0.49–1.00)	2.76 (0.74–10.23)	1.11 (0.80–1.53)	0.97 (0.82–1.13)	I^2^ = 24.2%, p = 0.228
Q3	0.94 (0.68–1.28)	0.91 (0.55–1.52)		0.90 (0.58–1.38)	0.98 (0.57–1.69)		0.84 (0.46–1.51)	0.84 (0.67–1.05)	0.54 (0.33–0.91)	1.24 (0.33–4.68)	0.84 (0.60–1.15)	0.83 (0.72–0.95)	I^2^ = 0.0%, p = 0.781

Adjusted for age at study entry (60–64 years, 65–69 years, 70–74 years, > 75 years), gender, smoking status (current smoker/former smoker/never smoker), and pack years (< 9 years, 9–17 years, 18–30 years, 31–46 years, > 47 years)

**Table 12:** Odds ratios of bladder cancer by total vegetable intake stratified by age < 60 years old

	Los Angeles BC ca–co study	Roswell Park Cancer Institute	Belgian ca–co study on BC	Stockholm ca–co study	New Hampshire BC study	Italian ca–co study on BC	Brescia BC study	NESCC	South and East China ca–co study	Molecular epidemiology of BC	Italian ca–co study	Pooled estimate	Test for heterogeneity
Total vegetables													
Q1	Ref	Ref	Ref	Ref	Ref	Ref	Ref	Ref	Ref	Ref	Ref	Ref	
Q2	0.97 (0.78–1.20)	0.70 (0.37–1.34)	0.61 (0.23–1.64)	1.51 (0.45–5.15)	0.82 (0.40–1.70)	0.50 (0.33–0.77)	0.85 (0.35–2.07)	1.08 (0.79–1.48)	0.62 (0.36–1.09)	1.09 (0.43–2.76)	1.08 (0.59–1.99)	0.80 (0.63–0.97)	I^2^ = 30.8%, p = 0.153
Q3	0.78 (0.62–0.97)	0.97 (0.47–2.02)	0.75 (0.30–1.83)	1.51 (0.47–4.87)	2.19 (1.02–4.69)	0.37 (0.24–0.57)	0.59 (0.22–1.56)	0.83 (0.59–1.18)	0.82 (0.47–1.43)	0.63 (0.26–1.54)	0.71 (0.39–1.29)	0.70 (0.52–0.88)	I^2^ = 47.8%, p = 0.038
Leaf vegetables													
Q1	Ref	Ref	Ref	Ref	Ref	Ref	Ref	Ref	Ref	Ref	Ref	Ref	
Q2	0.86 (0.70–1.07)	0.88 (0.47–1.64)	0.77 (0.30–1.98)	1.06 (0.34–3.24)	1.57 (0.74–3.32)	0.48 (0.24–0.96)	0.60 (0.24–1.50)	1.14 (0.82–1.58)	1.43 (0.83–2.45)	0.70 (0.27–1.80)	0.55 (0.32–0.96)	0.80 (0.64–0.97)	I^2^ = 24.0%. p = 0.215
Q3	0.70 (0.56–0.87)	0.55 (0.26–1.17)	0.76 (0.30–1.91)	0.56 (0.15–2.12)	2.37 (1.09–5.14)	0.51 (0.34–0.74)	1.55 (0.60–4.02)	0.89 (0.63–1.25)	1.32 (0.77–2.25)	0.55 (0.22–1.39)	0.98 (0.51–1.87)	0.70 (0.56–0.84)	I^2^ = 20.0%, p = 0.253
Shoot vegetables													
Q1	Ref	Ref	Ref	Ref	Ref		Ref	Ref	Ref		Ref	Ref	
Q2	1.06 (0.86–1.31)	0.99 (0.52–1.92)	0.68 (0.25–1.83)	2.30 (0.72–7.34)	1.16 (0.60–2.23)		1.13 (0.43–2.97)	1.13 (0.83–1.52)	1.01 (0.63–1.62)		1.09 (0.61–1.93)	1.06 (0.90–1.22)	I^2^ = 0.0%, p = 0.988
Q3	0.88 (0.70–1.09)	1.09 (0.53–2.26)	0.57 (0.22–1.51)	1.03 (0.31–3.36)	1.63 (0.55–4.86)		0.52 (0.21–1.30)	0.85 (0.55–1.32)	0.87 (0.43–1.75)		0.70 (0.38–1.29)	0.83 (0.68–0.97)	I^2^ = 0.0%, p = 0.910

Adjusted for age at study entry (< 45 years, 45–49 years, 50–54 years, 55–59 years), gender, smoking status (current smoker/former smoker/never smoker), and pack years (< 9 years, 9–17 years, 18–30 years, 31–46 years, > 47 years)

**Table 13:** Odds ratios of bladder cancer by total vegetable intake stratified by age ≥ 60 years old

	Los Angeles BC ca–co study	Roswell Park Cancer Institute	Belgian ca–co study on BC	Stockholm ca–co study	New Hampshire BC study	Italian ca–co study on BC	Brescia BC study	NESCC	South and East China ca–co study	Molecular epidemiology of BC	Italian ca–co study	Pooled estimate	Test for heterogeneity
Total vegetables													
Q1	Ref	Ref	Ref	Ref	Ref	Ref	Ref	Ref	Ref	Ref	Ref	Ref	
Q2	0.87 (0.61–1.24)	0.79 (0.48–1.31)	0.85 (0.50–1.46)	1.04 (0.67–1.62)	1.06 (0.61–1.85)	0.84 (0.62–1.15)	1.14 (0.61–2.12)	0.95 (0.76–1.19)	0.68 (0.46–1.02)	0.84 (0.22–3.21)	0.65 (0.47–0.89)	0.82 (0.72–0.92)	I^2^ = 0.0%, p = 0.714
Q3	0.64 (0.45–0.90)	0.91 (0.56–1.48)	0.98 (0.56–1.72)	0.94 (0.61–1.45)	0.80 (0.47–1.39)	0.68 (0.50–0.93)	1.44 (0.77–2.68)	1.01 (0.81–1.25)	0.89 (0.59–1.33)	1.17 (0.32–4.29)	0.77 (0.55–1.06)	0.81 (0.71–0.91)	I^2^ = 0.0%, p = 0.481
Leaf vegetables													
Q1	Ref	Ref	Ref	Ref	Ref	Ref	Ref	Ref	Ref	Ref	Ref	Ref	
Q2	0.75 (0.54–1.04)	0.60 (0.37–0.98)	0.66 (0.38–1.13)	0.76 (0.50–1.14)	0.74 (0.43–1.28)	0.76 (0.45–1.29)	0.56 (0.31–1.02)	1.16 (0.92–1.46)	1.40 (0.94–2.09)	1.06 (0.27–4.17)	0.88 (0.65–1.18)	0.81 (0.67–0.95)	I^2^ = 36.7%, p = 0.106
Q3	0.96 (0.68–1.37)	0.54 (0.33–0.88)	0.87 (0.49–1.53)	0.76 (0.47–1.23)	0.62 (0.36–1.05)	0.66 (0.49–0.88)	0.77 (0.40–1.49)	1.14 (0.91–1.43)	1.63 (1.06–2.50)	1.25 (0.35–4.41)	0.88 (0.62–1.27)	0.83 (0.67–0.99)	I^2^ = 49.3%, p = 0.032
Shoot vegetables													
Q1	Ref	Ref	Ref	Ref	Ref		Ref	Ref	Ref		Ref	Ref	
Q2	0.93 (0.66–1.29)	0.86 (0.53–1.38)	1.01 (0.59–1.72)	1.46 (0.95–2.25)	0.85 (0.52–1.40)		1.08 (0.58–2.00)	0.92 (0.75–1.13)	1.29 (0.90–1.86)		0.76 (0.55–1.05)	0.92 (0.81–1.04)	I^2^ = 0.0%, p = 0.538
Q3	0.80 (0.57–1.13)	0.77 (0.46–1.28)	1.01 (0.57–1.79)	1.19 (0.75–1.88)	0.98 (0.48–2.02)		1.27 (0.68–2.37)	0.87 (0.67–1.12)	1.04 (0.60–1.78)		0.87 (0.63–1.21)	0.89 (0.76–1.01)	I^2^ = 0.0%, p = 0.932

Adjusted for age at study entry (60–64 years, 65–69 years, 70–74 years, > 75 years), gender, smoking status (current smoker/former smoker/never smoker), and pack years (< 9 years, 9–17 years, 18–30 years, 31–46 years, > 47 years)
